# The Dimensional Structure of the Gambling Attitudes and Beliefs Survey: Challenging the Assumption of the Unidimensionality of Gambling-Specific Cognitive Distortions

**DOI:** 10.1007/s10899-022-10133-7

**Published:** 2022-05-28

**Authors:** Josefine Gehlenborg, Steffen Moritz, Lara Bücker

**Affiliations:** University Medical Centre Hamburg-Eppendorf, Hamburg, Germany

**Keywords:** Gambling, GABS, Cognitive biases, Irrational beliefs, Multidimensionality

## Abstract

The aim of the present study was to examine the dimensional structure of the Gambling Attitudes and Beliefs Survey (GABS). The GABS was administered to a sample of 415 individuals with self-reported problem or pathological gambling who were taking part in two different treatment studies preregistered with the German Clinical Trials Register (DRKS00013888) and ClinicalTrials.gov (NCT03372226). Exploratory factor analyses revealed a three-factor structure. We labeled the factors sensation seeking/illusion of control, luck/gambler’s fallacy, and attitude/emotions. Subsequent confirmatory factor analyses proved the three-factor model superior to the one-factor model proposed by the developers of the GABS. All dimensions were significantly correlated with symptom severity scores. Group comparisons showed significantly higher factor scores on the first factor (sensation seeking/illusion of control) for individuals reporting both skill-based and chance-based gambling compared to those reporting only chance-based gambling. The present study questions the unidimensionality of the GABS. A multidimensional assessment of gambling-related cognitive biases, beliefs, and positively valued attitudes may be useful in determining treatment outcomes and goals and in the development of novel interventions.

## Introduction

Pathological gambling is classified as a behavioral addiction (American Psychiatric Association, 2013) that entails severe psychosocial, financial, and legal impairments (Cowlishaw et al., 2016) as well as high psychiatric comorbidity, further increasing the distress of those affected (Kessler et al., 2008; Lorains et al., 2011). Even subclinical gambling results in a lower quality of life and an increased number of stressful live events compared to non-gambling individuals (Weinstock et al., 2017). Gambling-related cognitive biases and irrational beliefs are considered important mechanisms in the formation and maintenance of problem and pathological gambling (Ciccarelli et al., 2017; Cocker & Winstanley, 2015; Fortune & Goodie, 2012). The presence of these biases and beliefs has been found to increase the chance of relapse (Oei & Gordon, 2008; Smith et al., 2015), whereas a reduction has been found to predict recovery (Rossini-Dib et al., 2015). Therefore, the assessment and monitoring of gambling-related cognitive distortions is deemed important for treatment (Bodor et al., 2021).

Gambling-related cognitive distortions are frequently assessed with the Gambling Attitudes and Beliefs Survey (GABS; Breen & Zuckerman, 1994). The GABS is a 35-item self-report questionnaire capturing “a wide range of cognitive biases, irrational beliefs, and positively valued attitudes to gambling” (Breen & Zuckerman, 1999, p. 1102). Items are responded to on a 4-point Likert scale ranging from 1 = “strongly disagree” to 4 = “strongly agree.” Higher scores on the GABS indicate positive attitudes towards gambling (e.g., exciting, socially meaningful) and proneness to cognitive distortions regarding luck and strategies (e.g., illusion of control, gambler’s fallacy; Breen & Zuckerman, 1999). The GABS shows good internal consistency (Cronbach’s α = 0.90) and significant correlation with gambling severity and frequency (Breen & Zuckerman, 1999; Breen et al., 2001; Strong, Breen, et al., 2004).

The developers of the GABS, Breen and Zuckerman (1999), were surprised to find a unidimensional structure of the GABS capturing an overall *gambling affinity*. Unfortunately, in their original study they did not describe the results of their factor analyses in detail, and there has been disagreement in the literature on the dimensionality of the GABS ever since. For example, Strong and colleagues initially found that it had a two-factor structure but evaluated the second factor as uninterpretable and concluded that their findings lent further support to the unidimensionality of the measure (Strong, Breen, et al., 2004). Moreover, they proposed a 15-item short form of the GABS with comparable quality to the original 35-item version. In another study, they investigated the unidimensionality of their 15-item version with a confirmatory analysis and found a predominant first dimension plus two additional factors (Strong, Daughters, et al., 2004). However, they excluded five items, leaving a unidimensional 10-item version of the GABS, which they recommended for use with male college student gamblers, and did not further investigate or discuss the two additional factors that initially emerged in their analyses. Further questioning the unidimensionality of the GABS, Bouju and colleagues’ confirmatory factor analyses revealed a bad fit for the one-factor structure of the GABS in their data (Bouju et al., 2014). They proposed a 23-item version of the GABS with a five-factor structure (strategies, chasing, attitudes, luck, and emotions) with a significantly better fit.

In view of these inconsistent findings and the aforementioned limitations of previous studies, the aim of the present study was to reexamine the dimensional structure of the GABS in a sample of individuals with self-reported gambling problems. As the GABS-15 was found to be of equal psychometric quality to the original form (Strong, Breen, et al., 2004) and might be of greater use in gambling research due to its lower number of items, we focused on this short form of the GABS. As the presence of different cognitive distortions has been found to differ between individuals preferring different forms of gambling (e.g., the higher presence of illusion of control in skill-based vs. chance-based gambling; Kalke et al., 2018; Mallorquí-Bagué et al., 2019), we assumed that gambling-related cognitive distortions would be best represented by more than one latent construct and therefore expected a multidimensional structure of the GABS-15 to emerge.

## Methods

### Study Design

The present study used data from the baseline assessments of two randomized controlled trials (RCTs) on the effectiveness of unguided Internet-based interventions in problem and pathological gambling (Bücker et al., 2018, 2021; Wittekind et al., 2019). The first RCT examined the effectiveness of the depression-focused program Deprexis in 140 slot-machine gamblers with self-reported gambling and mood problems compared to a wait-list control group (Bücker et al., 2018). The second RCT examined the effectiveness of the gambling-specific program Restart (Bücker et al., 2021) compared to a wait-list control group and an Internet-based approach-avoidance task (AAT; Wittekind et al., 2019) compared to sham training in 265 participants with self-reported gambling problems. Deprexis and Restart are both based on cognitive-behavioral therapy and provide psychoeducational texts, videos, audios, and interactive exercises. Both RCTs incorporated two times of measurement (baseline and post intervention), which were enrolled online using the software Questback^®^.

On the first page of the baseline assessment, participants were informed about the aims and design of the particular trial. After providing informed consent for study participation, sociodemographic data and several questionnaires on symptom severity were applied. Finally, participants were asked to provide a pseudonymous e-mail address and create a personal code. Next, participants were randomly allocated to one of the intervention groups (direct access to either Deprexis, Restart, or AAT), or control groups (access to the intervention after completion of the post assessment) of the particular trial. Randomization was conducted using parallel assignment (ratio 1:1 or 1:1:1:1, respectively) and a computer-generated randomization sequence (block randomization). Participants were informed about group allocation via e-mail. For the intervention group, the e-mail included information on how to access Deprexis, Restart, or the AAT/sham training. After an intervention period of 8 weeks, participants were invited to the post assessment via e-mail. As an incentive, participants received a self-help manual about progressive muscle relaxation and mindfulness (Bücker et al., 2018; Wittekind et al., 2019) or an Amazon^®^ voucher of 20€ and access to an online approach bias modification training for gambling (Bücker et al., 2021).

The ethics committee of the German Society for Psychology assessed both RCTs as ethically unobjectionable (DGPs; Deprexis/AAT: SM 012014_2; Restart: SM 092017_amd_012014_2b). Moreover, both studies were preregistered with either the German Clinical Trials Register (Deprexis/AAT; registration number: DRKS00013888) or ClinicalTrials.gov (Restart; registration number: NCT03372226).

### Participants

Participants were recruited online via gambling- and addiction-related Internet forums, Facebook^®^ groups, and information websites. Moreover, a Google AdWords^®^ campaign was run in German-speaking countries that displayed the webpage of the studies to individuals entering keywords such as “treatment + gambling disorder” or “self-help + gambling” into the search engine Google^®^. The webpage of the studies contained information on study participation and the link to the baseline assessment. In addition, study flyers were sent to several institutions, including counseling centers and gambling halls.

Inclusion criteria for both studies were age between 18 and 65 (75 in the Restart-RCT), self-reported problem with pathological gambling and emotional distress (no formal diagnosis needed), Internet access, sufficient command of the German language, and informed consent. Exclusion criteria were acute suicidality (assessed with one item of the Patient Health Questionnaire-9 depression module) or a lifetime diagnosis of a psychotic disorder (assessed by self-report). Treatment as usual (TAU) was allowed for all participants in both studies.

### Questionnaires

Only the measures of the two RCTs that were used for data analysis in the present study are described here. In addition to the instruments described in this section, we assessed sociodemographic variables (e.g., age, gender) and gambling-related characteristics (e.g., age at first gambling, average monthly loss).

#### Gambling Attitudes and Beliefs Survey-15 (GABS-15)

Similar to its original form, the 15-item short version of the GABS (Strong, Breen, et al., 2004) measures irrational beliefs and attitudes towards gambling on a 4-point Likert scale (1 = “strongly disagree” to 4 = “strongly agree”). Higher scores indicate higher gambling affinity. The GABS-15 differentiates between non-problem, problem, and pathological gambling and demonstrates high incremental validity (Strong, Breen, et al., 2004).

#### Pathological Gambling Adaptation of the Yale-Brown Obsessive–Compulsive Scale (PG-YBOCS)

The PG-YBOCS (Pallanti et al., 2005) assesses previous-week gambling-related symptom severity. It contains 10 items on the two subscales *thoughts/urges* and *behavior.* The total score can range from zero to 40 and differentiates between subclinical (0–7), mild (8–15), moderate (16–23), severe (24–31), and extreme (32–40) gambling severity. Internal consistency is good for both the total score (Cronbach’s α = 0.97) and the subscales (Cronbach’s α = 0.93–0.94; Pallanti et al., 2005).

#### South Oaks Gambling Screen (SOGS)

The SOGS (Lesieur & Blume, 1987) uses 20 items to assess gambling severity over the previous 6 months. The total score can range from zero to 20 and differentiates between subclinical gambling (0–2), at-risk gambling (3–4). and pathological gambling (5–20). Internal consistency of the SOGS was found to be moderate (Cronbach’s α = 0.69), and convergent validity was found to be good (Goodie et al., 2013).

#### Patient Health Questionnaire-9 Depression Module (PHQ-9)

The PHQ-9 (Kroenke et al., 2001) has nine items assessing depressive symptoms experienced in the previous 2 weeks. Items can be answered on a 4-point Likert scale (“not at all” [0] to “almost every day” [3]). The total score can range from zero to 27 and differentiates between minimal (0–4), mild (5), moderate (10–14), and severe (15–27) symptomology. The internal consistency of the PHQ-9 is high (Cronbach’s α = 0.86–0.89; Kroenke et al., 2001).

### Statistical Analyses

All analyses were performed using IBM SPSS Statistics 27. We conducted varimax-rotated exploratory factor analyses with all items of the GABS-15. We used both the Kaiser-Guttman criterion and scree plot inspection for interpretation. Factor scores were saved to the dataset and correlated with scores on gambling-related and depressive symptom severity. To compare our factor solution to the unidimensional factor structure proposed by Breen and Zuckerman (1999), subsequent confirmatory factor analyses were conducted using the SPSS extension SPSS2LAVAAN. Chi-square test, comparative fit index (CFI), Tucker–Lewis index (TLI), root mean square error of approximation (RMSEA), and standardized root mean square residual (SRMR) were used for interpretation. Finally, we compared factor scores of individuals reporting both skill-based and chance-based gambling to those reporting only chance-based gambling by means of unpaired *t* tests.

## Results

### Baseline Characteristics

Sociodemographic characteristics, severity of gambling, and depressive symptoms as well as gambling-related characteristics of the sample (N = 415) are presented in Table 1. Almost three quarters of the sample were male. Participants on average reported moderate gambling severity and moderate depressive symptoms at baseline.

**Table 1:** Baseline characteristics of the sample (N = 415)

Sociodemographic variables	
Gender (male/female)	303/112 73.0%/27.0%
Age (in years)	35.24 (10.85)
Formal school education (in years)	10.76 (1.49)
Psychopathological data	
PG-YBOCS total score	18.67 (6.53)
PG-YBOCS behavior	9.23 (3.50)
PG-YBOCS thoughts	9.44 (3.45)
SOGS	10.15 (3.06)
PHQ-9	11.16 (5.16)
Gambling-related characteristics	
Age at first gambling (in years)	21.19 (8.86)
Age at frequent gambling (in years)	24.47 (9.19)
Monthly loss (in €)	994.21 (1189.57)
Debt (in €)	8398.85 (16,443.74)

Frequency and percentages or means and standard deviations (in brackets); Wittekind et al., 2019

*PG-YBOCS* Pathological Gambling Adaptation of the Yale-Brown Obsessive–Compulsive Scale, *PHQ-9* Patient Health Questionnaire-9 Depression Module, *SOGS* South Oaks Gambling Screen; Participants were not required to indicate their monthly loss (*n* = 380) or debts (*n* = 257)

### Factor Analysis

The matrix of intercorrelations of GABS items is displayed in Table 2. Criteria for conducting an exploratory factor analysis were met with a Kaiser–Meyer–Olkin index of 0.87, which confirmed sampling adequacy, and a significant Bartlett test (χ^2^(105) = 1972.43, *p* < 0.001), which confirmed the assumption of sphericity. According to the Kaiser-Guttmann criterion (eigenvalue > 1), three factors were extracted, which explained 53% of the total variance. Taking into account prior findings on the dimensionality of gambling-specific attitudes and beliefs (e.g., Bouju et al., 2014; Moritz et al., 2021; Steenbergh et al., 2002), we labeled the first dimension *sensation seeking/illusion of control* (explained variance after rotation: 20%), the second dimension *luck/gambler’s fallacy* (explained variance after rotation: 19%), and the third dimension *attitude/emotions* (explained variance after rotation: 14%). Factor loadings for all 15 items are presented in Table 3.

**Table 2:** Intercorrelations of GABS-15

Item	1	2	3	4	5	6	7	8	9	10	11	12	13	14	15
1. Gambling makes me feel really alive	1	.344	.302	.247	.305	.262	.315	.341	.183	.174	.249	.257	.201	.251	.277
2. If I have not won any of my bets for a while, I am probably due for a big win		1	.427	.203	.236	.245	.419	.299	.290	.165	.413	.239	.268	.355	.671
3. I know when I am on a streak			1	.144	.328	.214	.413	.224	.344	.215	.376	.243	.216	.388	.386
4. When I gamble, it is important to act as if I am calm even if I am not				1	.490	.171	.249	.160	.142	.415	.203	.258	.111	.216	.188
5. It is important to feel confident when I gamble					1	.241	.312	.213	.227	.396	.200	.343	.186	.297	.235
6. People who gamble are more daring and adventurous than those who never gamble						1	.309	.539	.338	.094	.221	.289	.248	.359	.330
7. Sometimes I just know I am going to have good luck							1	.405	.514	.126	.393	.364	.264	.455	.526
8. If you have never experienced the excitement of making a big bet, you have never really lived								1	.358	.162	.208	.346	.275	.376	.382
9. No matter what the game is, there are betting strategies that can help you win									1	.168	.240	.389	.359	.483	.430
10. If I lose at gambling, it is important to stay calm										1	.148	.266	.099	.159	.177
11. If I have been lucky lately, I should press my bets											1	.278	.252	.372	.435
12. I must be familiar with a gambling game if I am going to win												1	.269	.437	.339
13. Some people can bring bad luck to other people													1	.485	.346
14. To be successful at gambling, I must be able to identify streaks														1	.456
15. If I have lost my bets recently, my luck is bound to change															1

Item numbering refers to the original item order of the *GABS-15*

**Table 3:** Loadings of *GABS-15* items on the three dimensions from the varimax-rotated factor analysis

Item	Sensation seeking/illusion of control	Luck/gambler’s fallacy	Attitude/emotions
8. If you have never experienced the excitement of making a big bet, you have never really lived	**.744**		
6. People who gamble are more daring and adventurous than those who never gamble	**.729**		
9. No matter what the game is, there are betting strategies that can help you win	**.629**	.314	
14. To be successful at gambling, I must be able to identify streaks	**.610**	.404	
12. I must be familiar with a gambling game if I am going to win	**.538**		.363
13. Some people can bring bad luck to other people	**.533**		
2. If I have not won any of my bets for a while, I am probably due for a big win		**.792**	
15. If I have lost my bets recently, my luck is bound to change	.364	**.726**	
11. If I have been lucky lately, I should press my bets		**.682**	
3. I know when I am on a streak		**.656**	
7. Sometimes I just know I am going to have good luck	.458	**.550**	
4. When I gamble, it is important to act as if I am calm even if I am not			**.783**
5. It is important to feel confident when I gamble			**.745**
10. If I lose at gambling, it is important to stay calm			**.744**
1. Gambling makes me feel really alive		.335	.337

Loadings > .5 are set in bold type. Only factor loadings > .3 are presented. Items are ordered based on factor loadings. Item numbering refers to the original item order of the GABS-15

Six items loaded on the first dimension (all loadings > 0.5), four items loaded on the second dimension (all loadings > 0.6), and three items loaded on the third dimension (all loadings > 0.7). Two items showed ambiguous loadings (item 1: *Gambling makes me feel really alive*; item 7: *Sometimes I just know I am going to have good luck*). All factors showed small correlations with SOGS scores and PHQ-9 (see Table 4). Whereas attitude/emotions was not correlated with the PG-YBOCS total as well as subscale scores (all *p* > 0.05), sensation seeking/illusion of control was significantly correlated with the total score as well as the subscales thoughts and behavior at small effect sizes. For luck/gambler’s fallacy, moderate correlations were observed with the PG-YBOCS total score and the subscale thoughts, whereas small correlations were observed for the subscale behavior. None of the three factors correlated significantly with amount of monthly loss or debts (all *p* > 0.05). Only sensation seeking/illusion of control but not luck/gambler’s fallacy and attitude/emotions significantly correlated with age at first gambling, age at frequent gambling, and number of forms of gambling played.

**Table 4:** Correlation coefficients of the three extracted factors and psychopathological and gambling-related variables

Variable	Sensation seeking/illusion of control	Luck/gambler’s fallacy	Attitude/emotions
SOGS	.123*	.224***	.152**
PG-YBOCS total	.197***	.313***	.078
PG-YBOCS thoughts	.192***	.337***	.057
PG-YBOCS behavior	.177***	.252***	.089
PHQ-9	.142**	.184***	.127*
Amount of monthly loss (€)	.083	.083	.003
Debts (€)	.006	.005	.065
Age at first gambling (years)	–.157**	–.050	.052
Age at frequent gambling (years)	–.165**	–.042	.032
Number of forms of gambling played	.153**	.063	.018

*PG-YBOCS* Pathological Gambling Adaptation of the Yale-Brown Obsessive–Compulsive Scale, *PHQ-9* Patient Health Questionnaire-9 Depression Module, *SOGS* South Oaks Gambling Screen; *p < .05; **p < .01; ***p < .001; Participants were not required to indicate their monthly loss (*n* = 380) or debts (*n* = 257)

Interestingly, scree plot inspection indicated that the data could also be represented by only one factor, which is in line with the assumption of the unidimensionality of the GABS and had previously been proposed for the GABS and the GABS-15 (Breen & Zuckerman, 1999; Strong, Breen, et al., 2004). We compared our three-factor solution to a one-factor solution in confirmatory factor analyses. Items 1 and 7 were excluded from these analyses due to ambiguous loadings (see above). Confirmatory factor analyses proved the three-factor model (χ^2^(62) = 178.40, *p* < 0.001, CFI = 0.93, TLI = 0.91, RMSEA = 0.07, SRMR = 0.05) superior compared to the conventional one-factor model (χ^2^(65) = 434.70, *p* < 0.001, CFI = 0.76, TLI = 0.71, RMSEA = 0.12, SRMR = 0.08). However, the statistically significant chi square test of both models, which indicates that the observed and expected covariance matrices differ significantly, implies that none of the compared models provides an ideal fit for the present data. On the other hand, it should be noted that the chi square test in confirmatory factor analysis is affected by sample size, with *p* values considerably decreasing in larger samples (Alavi et al., 2020; Babyak & Green, 2010).

### Group Differences in Factor Loadings

Based on the first item of the SOGS, we categorized the forms of gambling played by participants as skill-based (i.e., card games, sports betting, horse betting, stock games, and skill games) and chance-based (i.e., dice games, casino games, lotteries, bingo, slot machines, and scratch lotteries). The majority of participants reported playing only chance-based games (*n* = 217); only nine participants reported playing only skill-based games. Playing both skill-based and chance-based forms of gambling was indicated by 183 participants. We then compared factor scores of individuals reporting chance-based gambling to those reporting skill-based gambling or both gambling forms. Unpaired *t* tests showed significantly higher scores on the first factor (sensation seeking/illusion of control) for the latter group (*t *(407) = 3.96, *p* < 0.001, *d* = 0.39). No group differences emerged regarding luck/gambler’s fallacy (*t *(407) = 1.47, *p* = 0.143, *d* = 0.15) or attitude/emotions (*t *(407) = 1.24, *p* = 0.216, *d* = 0.12).

## Discussion

The aim of the present study was to examine the dimensional structure of the GABS-15 in a sample of individuals with problem and pathological gambling. As hypothesized, a multidimensional structure emerged in the exploratory factor analysis, which is in line with a prior factor analysis challenging the unidimensional construct of the GABS (Bouju et al., 2014) and supports the statement of the scale’s developers, Breen and Zuckerman: “the fact that only one factor emerged was surprising” (Breen & Zuckerman, 1999, p. 1102). Three factors emerged, which we labeled sensation seeking/illusion of control, luck/gambler’s fallacy, and attitude/emotions. Subsequent confirmatory factor analyses found our three-factor model superior to the one-factor model.

In a follow-up step, we aimed to assess convergent external correlates for these factors in order to validate the multidimensional model. All factor scores were significantly positively correlated with symptom severity scores, with strongest effect sizes found for the second factor (luck/gambler’s fallacy). Moreover, significantly higher scores on the first factor (sensation seeking/illusion of control) were found for individuals playing both skill-based and chance-based games compared to those playing only chance-based games. These findings are in line with recent studies reporting greater illusion of control in individuals preferring skill-based forms of gambling such as poker or sports betting (Kalke et al., 2018; Mallorquí-Bagué et al., 2019) and further bring into question the relevance of illusion of control in chance-based forms of gambling such as slot machines (Moritz et al., 2021).

Our findings imply that cognitive distortions are represented by more than one latent construct. As proposed by Bouju and colleagues (2014), a multidimensional approach of assessing cognitive biases, irrational beliefs, and positively valued attitudes to gambling might be helpful as it would allow treatment to be tailored to the specific beliefs and biases experienced by specific patients. Moreover, a multidimensional assessment might also help to further investigate differences in cognitive distortions experienced by different subgroups of individuals with gambling problems and contribute to more tailored treatment approaches.

Importantly, it has to be noted that the sample of the present study was comprised of help-seeking individuals with subclinical and clinical gambling problems. Therefore, our findings are limited to individuals seeking help; they might be more aware of dysfunctional thoughts compared to individuals not seeking help. Moreover, it remains to be tested whether our factor structure can be replicated in a sample of individuals with pathological gambling with a verified diagnosis and in a sample of healthy individuals. Finally, as described in the results section of this paper, the model fit was not fully satisfactory for either the three-factor or the unidimensional model.

To conclude, our findings further question the unidimensionality of the GABS. A multidimensional assessment of gambling-related cognitive biases, beliefs, and positively valued attitudes may be informative, especially for planning treatment targets. Psychotherapy research should take into account the multidimensionality of biases when devising new interventions as not all individuals and not all games are prone to the same fallacies/biases.

## Data Availability

The data that support the findings of this study are available from the corresponding author upon request.
